# Venous return physiology applied to post-cardiac arrest haemodynamic management: a post hoc analysis of the NEUROPROTECT trial

**DOI:** 10.1186/s40635-024-00657-0

**Published:** 2024-08-13

**Authors:** Anders Aneman, Markus Benedikt Skrifvars, Koen Ameloot

**Affiliations:** 1 Intensive Care Unit, Liverpool Hospital, South Western Sydney Local Health District and South Western Sydney Clinical School, University of New South Wales, Sydney, Australia; 2 The Ingham Institute for Applied Medical Research, Sydney, Australia; 3 Faculty of Health Sciences, Macquarie University, Sydney, Australia; 4 Department of Emergency Care and Services, Helsinki University Hospital and University of Helsinki, Helsinki, Finland; 5 Department of Cardiology, Ziekenhuis Oost-Limburg, Genk, Belgium; 6 Departement de Cardiologie/Soins Intensifs Adultes, CHC-Montlégia, Liège, Belgique; 7 Department of Cardiology, University Hospitals Leuven, Louvain, Belgium; 8 Faculty of Medicine and Life Sciences, University Hasselt, Diepenbeek, Belgium

**Keywords:** Mean systemic filling pressure, Cardiac arrest, Haemodynamic management

## Abstract

**Background:**

The European Resuscitation Council 2021 guidelines for haemodynamic monitoring and management during post-resuscitation care from cardiac arrest call for an individualised approach to therapeutic interventions. Combining the cardiac function and venous return curves with the inclusion of the mean systemic filling pressure enables a physiological illustration of intravascular volume, vasoconstriction and inotropy. An analogue mean systemic filling pressure (*P*msa) may be calculated once cardiac output, mean arterial and central venous pressure are known. The NEUROPROTECT trial compared targeting a mean arterial pressure of 65 mmHg (standard) versus an early goal directed haemodynamic optimisation targeting 85 mmHg (high) in ICU for 36 h after cardiac arrest. The trial data were used in this study to calculate post hoc *P*msa and its expanded variables to comprehensively describe venous return physiology during post-cardiac arrest management. A general estimating equation model was used to analyse continuous variables split by standard and high mean arterial pressure groups.

**Results:**

Data from 52 patients in each group were analysed. The driving pressure for venous return, and thus cardiac output, was higher in the high MAP group (*p* < 0.001) along with a numerically increased estimated stressed intravascular volume (mean difference 0.27 [− 0.014–0.55] L, *p* = 0.06). The heart efficiency was comparable (*p* = 0.43) in both the standard and high MAP target groups, suggesting that inotropy was similar despite increased arterial load in the high MAP group (*p* = 0.01). The efficiency of fluid boluses to increase cardiac output was increased in the higher MAP compared to standard MAP group (mean difference 0.26 [0.08–0.43] fraction units, *p* = 0.01).

**Conclusions:**

Calculation of the analogue mean systemic filling pressure and expanded variables using haemodynamic data from the NEUROPROTECT trial demonstrated an increased venous return, and thus cardiac output, as well as increased volume responsiveness associated with targeting a higher MAP. Further studies of the analogue mean systemic filling pressure and its derived variables are warranted to individualise post-resuscitation care and evaluate any clinical benefit associated with this monitoring approach.

**Supplementary Information:**

The online version contains supplementary material available at 10.1186/s40635-024-00657-0.

## Background

The European Resuscitation Council 2021 guidelines for haemodynamic monitoring and management during post-resuscitation care from cardiac arrest state that perfusion should be maintained with fluids, noradrenaline and/or dobutamine depending on individual patient needs for intravascular volume, vasoconstriction or inotropy [1]. Multiple concurrent factors affect systemic perfusion in the post-cardiac arrest setting, emphasising the need for an adequate monitoring approach to guide therapy in individual patients. The venous return physiology proposed by Guyton [2] provides a physiologically robust framework to evaluate the principal components of cardiac performance, combining the venous return and cardiac function curves [3]. The pressure gradient between the mean systemic filling pressure and the right atrial pressure represents the driving pressure for venous return, and thus determines the cardiac output [4]. The mean systemic filling pressure is the result of the elastic recoil pressure of the intravascular volume during no-flow conditions, or more precisely the stressed intravascular volume divided by the averaged systemic vascular compliance [5, 6]. Several methods exist to estimate the mean systemic filling pressure in the clinical setting [7]. The mathematical model developed by Parkin and Leaning to calculate an analogue mean systemic filling pressure (*P*_msa_) [8] has been experimentally validated [9–11] and is best aligned with measurements in humans [12–14]. Haemodynamic monitoring in post-cardiac arrest patients often include mean arterial pressure (MAP), central venous pressure (CVP) and cardiac output (CO) making the calculation of *P*_msa_ feasible. Once *P*_msa_ is known, several variables to evaluate venous return, cardiac function and the intravascular volume state may be derived. The application of measurable venous return physiology to evaluate haemodynamic post-resuscitation management has been very sparsely reported in the literature, with only one recent experimental study published [15]. In contrast, several studies of venous return physiology based on *P*_msa_ have been reported in postoperative cardiac patients [16–21], during anaesthesia [22–24] and intensive care [25–27], and in a review of the passive leg raising test to assess volume responsiveness [28].

This exploratory study utilised data previously collected in a randomised study of goal-directed haemodynamic optimisation compared to standard care (the NEUROPROTECT trial [29, 30]) in post-cardiac arrest patients, applying *P*_msa_ and its derived variables to illustrate the venous return physiology. The aim was to use these variables to comprehensively describe the effects of volume administration, vasopressor and inotropic support in two different strategies of post-resuscitation haemodynamic management.

## Methods

The original data were captured in the randomised NEUROPROTECT clinical trial (NCT02541591). The protocol [30], cardiac arrest characteristics and primary outcomes [29] have been published earlier and this study is an exploratory, post hoc analysis of venous return physiology centred on *P*_msa_ in the haemodynamic management of comatose survivors of cardiac arrest. The study is reported according to the Strengthening the Reporting of Observational Studies in Epidemiology (STROBE) statement [31] (see Additional file 1).

The details of patient characteristics, the primary outcome of the extent of anoxic brain damage on diffusion-weighted magnetic resonance imaging, and the secondary outcomes of favourable neurological status at ICU discharge and at 180 days have been published [29]. In summary, comatose survivors of out-of-hospital cardiac arrest of a presumed cardiac cause were randomised to early goal directed haemodynamic optimisation (EGDHO) targeting a mean arterial pressure (MAP) of 85–100 mmHg and a mixed venous oxygen saturation (SvO2) of 65–75%, or to receive standard care with MAP maintained at 65 mmHg (MAP65) from admission to ICU and over the next 36 h. In the EGDHO group, fluids up to 3 L/24 h were administered as guided by stroke volume variation or a passive leg raising manoeuvre with blood transfusions used to maintain haemoglobin > 100 g/L, and infusions of dobutamine and/or norepinephrine added if necessary to achieve the haemodynamic targets. In the MAP65 group, haemodynamic support was provided at the discretion of the clinical team. Standard care in both groups included sedation (propofol, remifentanil), mechanical ventilation and targeted hypothermia at 33 °C for the first 24 h followed by rewarming at 0.3 °C/h until 36 °C.

### Venous return physiological variables

The application of venous return physiological variables to interpret the haemodynamic status of patients admitted to ICU has been detailed in previous reports, for example [17, 25, 28], and the concepts are illustrated in the Additional File 2. *P*_msa_ was calculated according to Parkin and Leaning [8]:

$$ P_{{{\text{msa}}}} = {\text{CVP}} \cdot 0.{96 } + {\text{ MAP}} \cdot 0.0{4 } + {\text{ CO}} \cdot {\text{c}}\quad \quad \left( {{\text{mmHg}}} \right) $$

where the constant c incorporates the venoarterial compliance ratio and estimated venous resistance adjusted for age, height and weight [32]. The CO must equal the venous return (VR) to the heart which is regulated by the pressure gradient for venous return (VRdP) between *P*_msa_ and the right atrial pressure, represented by CVP. Together with the resistance to venous return (RVR), the CO is determined by

$$ {\text{CO}} = {\text{VR}} = \left( {P_{{{\text{msa}}}} - {\text{CVP}}} \right)/{\text{RVR}}\quad \quad \left( {{\text{L}}/{\text{min}}} \right) $$

The resistance to venous return (RVR) is hence calculated as the VRdP over CO. The pumping action of the heart maintains the VRdP, and the efficiency of the heart (*E*_h_) may thus be calculated as [8, 33]

$$ E_{{\text{h}}} = \left( {P_{{{\text{msa}}}} - {\text{CVP}}} \right)/P_{{{\text{msa}}}} \quad \quad \left( {0 \le E_{{\text{h}}} \le {1}} \right) $$

where a value ~ 1 reflects a normal heart function with CVP close to 0 and a value of 0 is seen in circulatory standstill when CVP and *P*_msa_ are equilibrated. In addition to the static *E*_h_, the degree to which a volume bolus changes the pumping capacity of the heart is defined by the ratio of the change in driving pressure for VR to the change in *P*_msa_, providing a dynamic measure of volume efficiency (*E*_vol_) [16, 17, 19]:

$$ E_{{{\text{vol}}}} = \Delta {\text{VRdP}}/\Delta P_{{{\text{msa}}}} \quad \quad \left( {0 \le E_{{{\text{vol}}}} \le {1}} \right) $$

An integrative measure of hydraulic pumping ability of the heart is represented by the product of MAP and CO, referred to as cardiac power, which correlates with clinical outcomes in cardiac patients [34–36]. The cardiac power (CP) was scaled to the volume state represented by *P*_msa_ as [16, 17, 19]:

$$ {\text{CP}} = \left( {[{\text{MAP}} \cdot {\text{CO}}]/{451}} \right)/P_{{{\text{msa}}}} \quad \quad \left( {{\text{W}}/{\text{mmHg}}} \right) $$

and further analysed as a dynamic variable following a fluid bolus by the change in CP over the change in *P*_msa_, referred to as cardiac power efficiency (*E*_CP_) similar to *E*_h_ and *E*_vol_. In addition preload and pump function variables, it is important to consider the external opposition to ventricular ejection represented by the arterial load. An integrative measure of cardiac afterload that includes steady and pulsatile components is given by the effective arterial elastance (Ea) calculated as [37, 38]

$$ {\text{Ea}} = 0.{9} \cdot {\text{systolic arterial pressure}}/{\text{stroke volume}}\quad \quad \left( {{\text{mmHg}}/{\text{mL}}} \right) $$

Finally, the stressed volume was derived from extrapolating the slope of the line connecting *P*_msa_ before and after a fluid bolus of 500 mL to the *x*-axis intercept corresponding to a *P*_msa_ = 0 [17, 20], with the assumption that the administered volume remained in the intravascular compartment from the start of infusion to the next measurement (30–40 min).

The potential confounding introduced by mechanical circulatory support on the equations for venous return physiology is unclear, and these patients were excluded in the analyses.

### Statistical analyses

Data are reported as means ± standard deviation or medians and interquartile ranges [IQR, 25th–75th percentile] as appropriate for variables with normal or non-normal distributions as judged by inspection of the Q–Q plots, Levene’s test of equality of variances, and the Shapiro–Wilk normality test. Categorical data are reported as counts and percentages. Longitudinal haemodynamic data were analysed using a generalised estimating equations (GEE) model that included factors for time, treatment and their interaction. The model included an exchangeable working correlation matrix to account for within-patient correlations. A Gaussian distribution with log-link function was used for all variables, except for *E*_h_, *E*_vol_, *E*_CP_ and Ea that were analysed using a gamma distribution since these variables were positive only and skewed to the right. Longitudinal haemodynamic data are reported as the marginal means and their 95% confidence intervals. The Wald test was applied to the robust standard errors in the GEE models and the robust *z* value reported as a *p* value from a normal distribution of the test statistic. In addition to the GEE model, data for the first 6 h were compared to the last 6 h using the Welch two sample *t* test with changes reported as mean difference including the 95% confidence interval. Correlations were assessed by Spearman’s correlation coefficient, rho, including the 95% confidence interval. Physiologically impossible data were deleted and similar to any missing data imputed using multiple imputation by chained equations with ten iterations, with the results from the calculated variables in each iteration pooled. Statistical analyses were performed using the R statistical software (v.4.0.3, R Foundation for Statistical Computing, Vienna, Austria) in RStudio using the ‘dplyr’, ‘mice’, ‘geepack’, ‘emmeans’ and ‘ggplot2’ packages. Statistical significance was set at a two-sided *p* < 0.05.

## Results

A total of 104 patients were included in this post hoc study with 52 patients in the EGDHO group and 52 patients in the MAP65 group. Patient and characteristics are presented in Table 1. One patient in each group was supported with intra-aortic balloon counterpulsation and excluded in the analyses. There were no differences in the fluid boluses administered with 28 patients given 54 boluses in MAP65 group, and 21 patients given 46 boluses in the EGDHO group. A similar and small number of patients received blood transfusions. Patients in the EGDHO group received more noradrenaline and dobutamine, but less propofol and with no difference in the dose of remifentanil (Table 1).

**Table 1:** Patient and treatment characteristics

	MAP65 (*n* = 52)	EGDHO (*n* = 52)	*p* value
Age (years)	65 ± 10	64 ± 8.1	0.63
Height (cm)	176 ± 8.0	172 ± 7.8	0.28
Weight (kg)	81 ± 10	78 ± 12	0.24
Gender (male)	39 (75%)	40 (77%)	0.82
IABP (patients)	1 (2%)	1 (2%)	0.14
Fluid bolus (patients)	29 (56%)	26 (50%)	0.56
Blood transfusion (patients)	2 (4%)	4 (8%)	0.40
Noradrenaline (mcg/kg/min)			
Cumulative	1.85 [0.65–4.4]	3.3 [1.11–6.95]	< 0.001
Time-weighted average	0.14 [0.06–0.19]	0.22 [0.12–0.38]	< 0.001
Dobutamine (mcg/kg/min)			
Cumulative	0 [0–28]	0 [0–40]	< 0.001
Time-weighted average	0 [0–1.66]	0 [0–2.49]	< 0.001
Propofol (mg/kg/min)			
Cumulative	0.23 [0.10–0.49]	0.17 [0.08–0.35]	< 0.001
Time-weighted average	0.017 [0.009–0.030]	0.011 [0.006–0.022]	< 0.001
Remifentanyl (mcg/kg/min)			
Cumulative	2.22 [1.04–3.75]	2.25 [1.12–3.48]	0.80
Time-weighted average	0.15 [0.1–0.15]	0.15 [0.1–0.15]	0.87

**Fig. 1:**
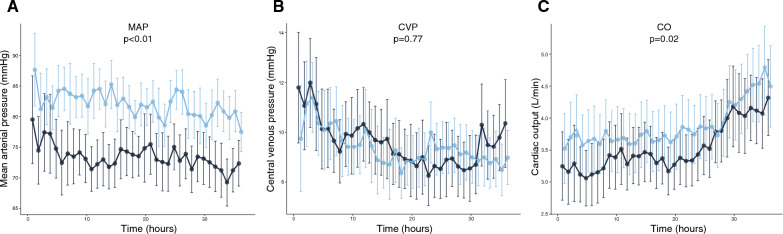
Changes in standard haemodynamic variables. Values are shown for the study period of 36 h from admission to ICU as marginal means and the 95% confidence intervals. **A** MAP, mean arterial pressure; **B** CVP, central venous pressure; **C** CO, cardiac output

The MAP was consistently higher in EGDHO compared to MAP65 (*p* < 0.01, Fig. 1A). There was no significant difference in CVP between groups (*p* = 0.77, Fig. 1B), while CVP decreased over time in both groups (mean difference 1.4 [0.4–2.3] mmHg, *p* = 0.004, in MAP65 and mean difference 1.9 [1.1–2.7] mmHg, *p* < 0.001, in EGDHO). The CO was higher in the EGDHO group (*p* = 0.02, Fig. 1C) with an increase over time in both groups (mean difference 0.60 [0.28–0.92] L/min, *p* < 0.001, in MAP65 and mean difference 1.13 [0.82–1.45] L/min, *p* < 0.001, in EGDHO).

**Fig. 2:**
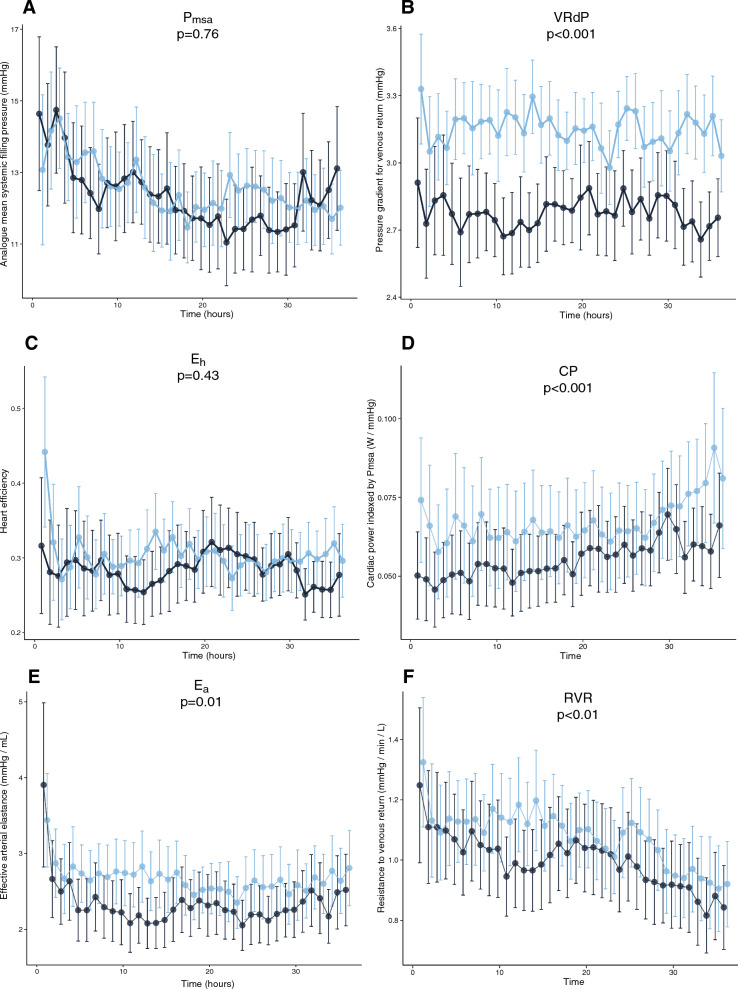
Changes in analogue mean systemic filling pressure and derived variables. Values are shown for the study period of 36 h from admission to ICU as marginal means and the 95% confidence intervals. **A** *P*_msa_, analogue mean systemic filling pressure; **B** VRdP, driving pressure for venous return; **C** *E*_h_, heart efficiency; **D** CP, cardiac power indexed by Pmsa; ***E*** Ea, effective arterial elastance; ***F*** RVR, resistance to venous return

*P*_msa_ was not different between groups (*p* = 0.76) but decreased over time (mean difference 1.88 [1.11–2.66] mmHg, *p* = 0.005, in MAP65 and mean difference 1.30 [0.38–2.21] mmHg, *p* < 0.001, in EGDHO) (Fig. 2A). The VRdP was higher in the EGHDO group (*p* < 0.001), consistent with the higher CO, with no significant changes over time (mean difference 0.02 [− 0.14–0.11] mmHg, *p* = 0.80, in MAP65 and mean difference 0.00 [− 0.11–0.12] mmHg, *p* = 0.90, in EGDHO) (Fig. 2B). *E*_h_ was not different between groups (*p* = 0.43) and did not change over time (mean difference 0.03 [− 0.01–0.06] mmHg, *p* = 0.10, in MAP65 and mean difference 0.02 [− 0.02–0.05] mmHg, *p* = 0.30, in EGDHO) (Fig. 2C). An increased *E*_vol_ (mean difference 0.26 [0.08–0.43], *p* = 0.01) for all bolus episodes was observed in the EGDHO group (0.42 [0.23–1.0]) compared to the MAP65 group (0.11 [0.02–0.89]).

The CP was greater in the EGDHO group (*p* < 0.001) with an increase over time observed in the EGDHO group (mean difference 0.012 [0.004–0.024) W/mmHg, *p* = 0.04] as well as in the MAP65 group (mean difference 0.015 [0.008–0.021 W/mmHg, *p* < 0.001) (Fig. 2D). *E*_CP_ was greater in the EGDHO group for all fluid boluses (0.17 [0.10–0.39]) compared to MAPG65 (0.08 [0.01–0.17]), *p* = 0.04.

*E*_a_ was greater in the EGDHO group (*p* = 0.01) with a decrease over time only observed in the MAP65 group (mean difference 0.43 [0.11–0.75] mmHg/mL, *p* = 0.01) (Fig. 2E). *E*_a_ decreased after fluid bolus administration in all patients (mean difference 0.34 [0.23–0.45] mmHg/mL, *p* < 0.001) but with no significant difference between the treatment groups. *E*_a_ correlated with the RVR (0.81 [0.80–0.83], *p* < 0.001) and SVR (0.81 [0.80–0.83], *p* < 0.001) in all patients. The RVR was higher in the EGDHO group (*p* < 0.01) and decreased over time in both EGDHO (0.18 [0.09–0.28] mmHg/min/L, *p* < 0.001) and MAP65 (0.26 [0.16–0.36] mmHg/min/L, *p* < 0.001) groups (Fig. 2F).

The stressed volume estimated from all bolus episodes during the study period was numerically greater in the EGDHO group (1.75 [1.43–2.04] L) compared to the MAP65 group (1.43 [0.72–1.82] L), although this difference did not attain statistical significance (mean difference 0.27 [− 0.014–0.55], *p* = 0.06).

## Discussion

The main findings of this explorative post-hoc study of the NEUROPROTECT haemodynamic data were: (1) the analogue mean systemic filling pressure reflecting the intravascular volume state was within a normal range, while decreasing over the study period up to 36 h after cardiac arrest; (2) the driving pressure for venous return, and thus cardiac output, was higher in the group targeting a higher MAP post-resuscitation along with an increased estimated stressed intravascular volume; (3) the heart efficiency was comparable in both the standard and high MAP target groups suggesting that inotropy was similar; (4) the cardiac power was higher in the group targeting a higher MAP as a result of maintaining both higher MAP and cardiac output; (5) the efficiency of fluid boluses to increase cardiac output and power was increased in the higher MAP compared to standard MAP group; and (6) both the arterial load and the resistance to venous return were increased in the higher MAP group while still sustaining an increased cardiac output.

*P*_msa_ over the first few hours following admission to ICU was similar to *P*_msa_ reported in postoperative cardiac patients [17] and patients with cardiogenic acute circulatory failure [25]. A decrease in *P*_msa_ was subsequently observed that is likely to reflect the receding vasoconstrictive effects of acute resuscitation drugs including adrenaline and vasodilation from the institution of sedation in ICU. An absolute reduction in intravascular volume seems less likely given the similar incidence of fluid boluses in both groups. While *P*_msa_ was not statistically significantly different between the two groups, it was numerically increased in the EGDHO with a less pronounced decrease over time.

A central finding of this study is the increased VRdP observed in the EGDHO group that sustained a greater cardiac output. This difference in VRdP was the combined result of the *P*_msa_ trending higher and the CVP trending lower in the EGDHO group. A composite variable like VRdP contributes more information on the volume and compliance state of the cardiovascular system than the isolated values of *P*_msa_ or CVP. The static nature of those latter variables limits any inference to vascular volume. This is often reported for CVP [39] but applies to *P*_msa_ as well, while this acknowledgement should not be construed to dismiss their combined validity [33]. The VRdP around 3 mmHg in both groups was at the lower end of the normal range of 3–8 mmHg reported in humans [12, 40, 41], and corresponded to a cardiac output less than 4 L/min for most of the duration of the study. An increased VRdP in the EGDHO group is also supported by the increased, although statistically not significantly different, stressed volume in this group compared to MAP65. The estimated 1.7 L in the EGDHO group is similar to previous reports during hypothermic circulatory arrest [42] and in postoperative cardiac patients [17, 20]. It should be noted that the stressed volume was estimated by extrapolation of *P*_msa_ changes before and after the administration of a fluid bolus. The number of bolus episodes were limited and dispersed over time. Thus caution is warranted when interpreting simple relationships between stressed volume estimates and *P*_msa_ or VRdP. Supporting a greater stressed volume and VRdP in the EGDHO group overall is the corresponding increase in *E*_vol_, meaning that the 500 ml fluid boluses when given in this group more effectively increased VRdP and hence cardiac output. The increased dosing of noradrenaline in the EGDHO group plausibly mobilised volume from the unstressed compartment of predominantly large splanchnic veins [43] to the stressed portion of the intravascular volume, by reducing compliance in these capacitance vessels.

*E*_h_ was not significantly different between the two groups and hence the increased cardiac output in the EGDHO group would not appear to be related to an increased pump efficiency of the heart. Targeting a higher MAP could in theory have improved the coronary perfusion pressure with additional inotropic support from dobutamine, although low doses were used. The increased CP in the EGDHO group was proportionally driven more by the higher MAP than the CO, while the increased *E*_CP_ reflected the increased cardiac output response to a volume bolus. The three efficiency variables are useful as they provide a scalar, continuous measure within the interval 0 to 1, as opposed to the frequently used dichotomous description of patients being ‘responsive’ or ‘non-responsive’ to haemodynamic interventions [19, 25]. Viewing *E*_h_, *E*_vol_ and *E*_CP_ together, the main effects of the EGDHO interventions were an increased responsiveness of cardiac output to the volume state.

The arterial load was increased in the EGDHO group as indicated by *E*_a_ and RVR variables, while no adverse cardiac effects from high MAP were reported in the original study [29] or in a subsequent meta-analysis [44]. The increased *E*_a_ is not unexpected given a higher MAP target in the EGDHO and it still represents a relatively schematic measurement of a complex cardiovascular phenomenon [37]. The greater RVR in the EGDHO group targeting a higher MAP was associated with vasoconstriction by the increased dose of noradrenaline and reduced vasodilation associated with the decreased dose of propofol. The decreased dose of propofol in the EGDHO group may also have facilitated reaching the target variables by reducing the effects of decreased cardiac output, myocardial depression and venodilation as reflected by increased VRdP in this group. The correlation between *E*_a_ and RVR arguably incorporates a degree of mathematical coupling from the inclusion of cardiac output or stroke volume in both variables. The arterial load represented by *E*_a_ is different from the average resistance to a blood corpuscular element represented by RVR.

This study has some important strengths and limitations. The detailed haemodynamic evaluation using venous return physiology adds novel insights into post-resuscitation management. The individualised approach to fluids, vasopressors and inotropes called for in the ERC 2021 guidelines [1] may be facilitated by the comprehensive set of continuous variables reported in this study, allowing an analysis of the main cardiovascular domains relating to intravascular volume, vascular resistance and heart pump function. The consecutive and granular data over 36 h of post-resuscitation management allowed cardiovascular dynamics to be assessed in detail. The study is limited by its post-hoc nature with potential residual confounding and the results should be viewed as exploratory and hypothesis generating. The precision of cardiac output monitoring in the MAP65 (Vigileo, Edwards Life Sciences) and the EGDHO (pulmonary artery catheter thermodilution) groups may vary but is unlikely to have had a major impact given the scaling of CO in the *P*_msa_ equation. Two tertiary-level hospitals contributed data to the original study, but the cohort is relatively small and its external validity may be challenged. Most data were obtained during hypothermia that may affect vascular tone and compliance and observations might be different in normothermic conditions.

In conclusion, applying venous return physiology to the analysis of post-resuscitation haemodynamic data in the NEUROPROTECT trial demonstrated an increased venous return and volume responsiveness associated with targeting a higher MAP improved systemic perfusion. Further studies of the analogue mean systemic filling pressure and its derived variables are warranted to individualise post-resuscitation care and evaluate any clinical benefit associated with this monitoring approach.

### Supplementary Information

Additional file 1. STROBE statement.

Additional file 2. Illustration of haemodynamic physiology concepts. **A** Cardiac output is determined by the intersection of the venous return and cardiac function curves, indicated in the graph for the positions pre and post the administration of a volume bolus. The corresponding change in venous return pressure gradient (VRdP) over the change in the analogue mean systemic filling pressure (P_msa_) describes the efficiency of the volume bolus to increase cardiac output (E_vol_). **B** Volume pressure curve of the left ventricle indicating the slope of the effective arterial elastance given by the end-systolic blood pressure (ESP) over the stroke volume (SV). **C** Analogue mean systemic filling pressure before (Pre P_msa_) administration of a known quantity of volume (500 mL) and the resulting increase in filling pressure (Post P_msa_). The line connecting these two observations was extrapolated to a P_msa_ of zero, indicating the stressed volume, i.e., the volume that starts to generate an elastic recoil pressure within the vasculature

## Data Availability

The data analysed for this post hoc analysis are not publicly available but are available from Dr. K Ameloot on reasonable request and pending approval by relevant ethics committees.

## Abbreviations

CO: Cardiac output
CVP: Central venous pressure
CP: Cardiac power
*E*_a_: Effective arterial elastance
*E*_CP_: Cardiac power efficiency
EGDHO: Early goal directed haemodynamic optimisation targeting MAP 85 mmHg
*E*_h_: Heart efficiency
*E*_vol_: Volume efficiency
MAP: Mean arterial pressure
MAP65: Standard haemodynamic therapy targeting MAP 65 mmHg
*P*_msa_: Analogue mean systemic filling pressure
RVR: Resistance to venous return
VRdP: Venous return pressure gradient
