# Supplementary material for: Venous return physiology applied to post-cardiac arrest haemodynamic management: a post hoc analysis of the NEUROPROTECT trial
Source: Intensive Care Med Exp. 2024 Aug 13;12:70. doi: 10.1186/s40635-024-00657-0 (PMC11322455; doi:10.1186/s40635-024-00657-0)
Supplement: Supplementary file 1 — Additional file 1. STROBE statement. [file 40635_2024_657_MOESM1_ESM.docx]

**Additional File 1.**

**Table S1.** STROBE Statement.

|  | Item No | Recommendation | Page No |
| --- | --- | --- | --- |
| **Title and abstract** | 1 | (*a*) Indicate the study’s design with a commonly used term in the title or the abstract | 1 |
|  |  | (*b*) Provide in the abstract an informative and balanced summary of what was done and what was found | 2-3 |
| Introduction | | | |
| Background/rationale | 2 | Explain the scientific background and rationale for the investigation being reported | 3-4 |
| Objectives | 3 | State specific objectives, including any prespecified hypotheses | 4 |
| Methods | | | |
| Study design | 4 | Present key elements of study design early in the paper | 5 |
| Setting | 5 | Describe the setting, locations, and relevant dates, including periods of recruitment, exposure, follow-up, and data collection | 5 (refs 29,30) |
| Participants | 6 | (*a*) Give the eligibility criteria, and the sources and methods of selection of participants. Describe methods of follow-up | 5 (refs 29,30) |
|  |  | (*b*) For matched studies, give matching criteria and number of exposed and unexposed |  |
| Variables | 7 | Clearly define all outcomes, exposures, predictors, potential confounders, and effect modifiers. Give diagnostic criteria, if applicable | 5 |
| Data sources/ measurement | 8* | For each variable of interest, give sources of data and details of methods of assessment (measurement). Describe comparability of assessment methods if there is more than one group | 6-8 |
| Bias | 9 | Describe any efforts to address potential sources of bias | refs 29,30 |
| Study size | 10 | Explain how the study size was arrived at | refs 29,30 |
| Quantitative variables | 11 | Explain how quantitative variables were handled in the analyses. If applicable, describe which groupings were chosen and why | NA |
| Statistical methods | 12 | (*a*) Describe all statistical methods, including those used to control for confounding | 8-9 |
|  |  | (*b*) Describe any methods used to examine subgroups and interactions |  |
|  |  | (*c*) Explain how missing data were addressed |  |
|  |  | (*d*) If applicable, explain how loss to follow-up was addressed |  |
|  |  | (*e*) Describe any sensitivity analyses |  |
| Results | | |  |
| Participants | 13* | (a) Report numbers of individuals at each stage of study—eg numbers potentially eligible, examined for eligibility, confirmed eligible, included in the study, completing follow-up, and analysed | 9 |
|  |  | (b) Give reasons for non-participation at each stage |  |
|  |  | (c) Consider use of a flow diagram |  |
| Descriptive data | 14* | (a) Give characteristics of study participants (eg demographic, clinical, social) and information on exposures and potential confounders | 9, Table 1 |
|  |  | (b) Indicate number of participants with missing data for each variable of interest |  |
|  |  | (c) Summarise follow-up time (eg, average and total amount) |  |
| Outcome data | 15* | Report numbers of outcome events or summary measures over time | 9-11 |

| Main results | 16 | (*a*) Give unadjusted estimates and, if applicable, confounder-adjusted estimates and their precision (eg, 95% confidence interval). Make clear which confounders were adjusted for and why they were included | 9-11 |
| --- | --- | --- | --- |
|  |  | (*b*) Report category boundaries when continuous variables were categorized |  |
|  |  | (*c*) If relevant, consider translating estimates of relative risk into absolute risk for a meaningful time period |  |
| Other analyses | 17 | Report other analyses done—eg analyses of subgroups and interactions, and sensitivity analyses | NA |
| Discussion | | | |
| Key results | 18 | Summarise key results with reference to study objectives | 11 |
| Limitations | 19 | Discuss limitations of the study, taking into account sources of potential bias or imprecision. Discuss both direction and magnitude of any potential bias | 14 |
| Interpretation | 20 | Give a cautious overall interpretation of results considering objectives, limitations, multiplicity of analyses, results from similar studies, and other relevant evidence | 15 |
| Generalisability | 21 | Discuss the generalisability (external validity) of the study results | 14-15 |
| Other information | | | |
| Funding | 22 | Give the source of funding and the role of the funders for the present study and, if applicable, for the original study on which the present article is based | 16 |

**Table S2.** Correlations (Spearman’s rho) between the dose of dobutamine and noradrenaline and the venous return physiology variables at the time points up to 36 hours.

|  | Dobutamine dose | p-value | Noradrenaline dose | p-value |
| --- | --- | --- | --- | --- |
| VRdP | 0.07 [0.04 – 0.09] | <0.001 | 0.06 [0.02 – 0.10] | <0.001 |
| CP | 0.09 [0.06 – 0.12] | <0.001 | 0.05 [0.03 – 0.08] | 0.003 |
| E_CP_ | 0.28 [0.13 – 0.46] | <0.001 | 0.15 [-0.09 – 0.37] | 0.14 |
| E_a_ | 0.06 [0.04 – 0.09] | <0.001 | 0.08 [0.05 – 0.10] | <0.001 |
| RVR | 0.06 [0.03 – 0.09] | <0.001 | 0.18 [0.15 – 0.20] | <0.001 |

VRdP = pressure gradient for venous return; CP = cardiac power; E_CP_ = cardiac power efficiency; E_a_= effective arterial elastance; RVR = resistance to venous return
