# Supplementary figures and images for: Venous return physiology applied to post-cardiac arrest haemodynamic management: a post hoc analysis of the NEUROPROTECT trial
Source: Intensive Care Med Exp. 2024 Aug 13;12:70. doi: 10.1186/s40635-024-00657-0 (PMC11322455; doi:10.1186/s40635-024-00657-0)

A

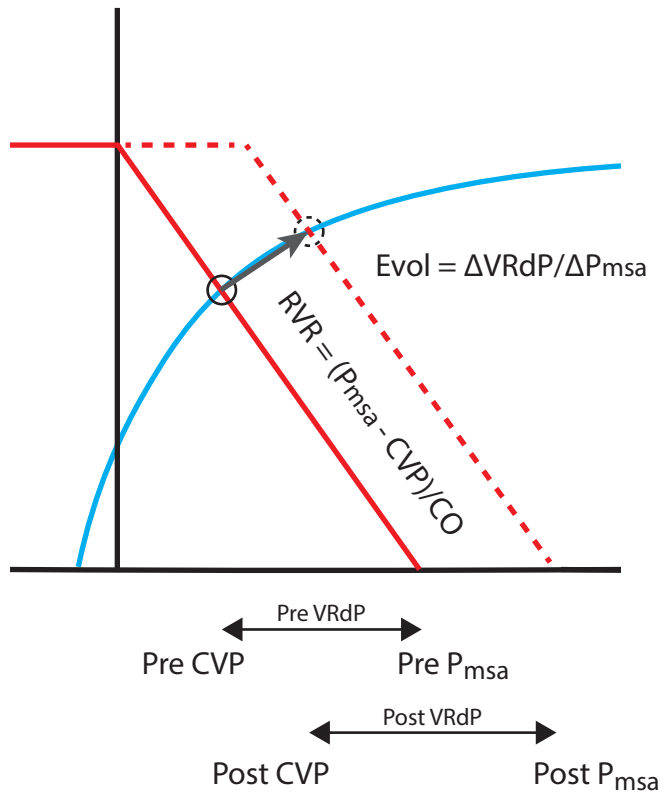

B

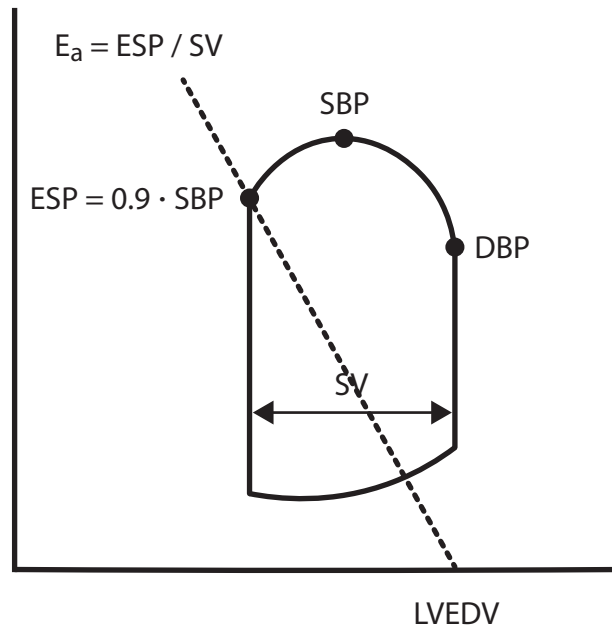

C

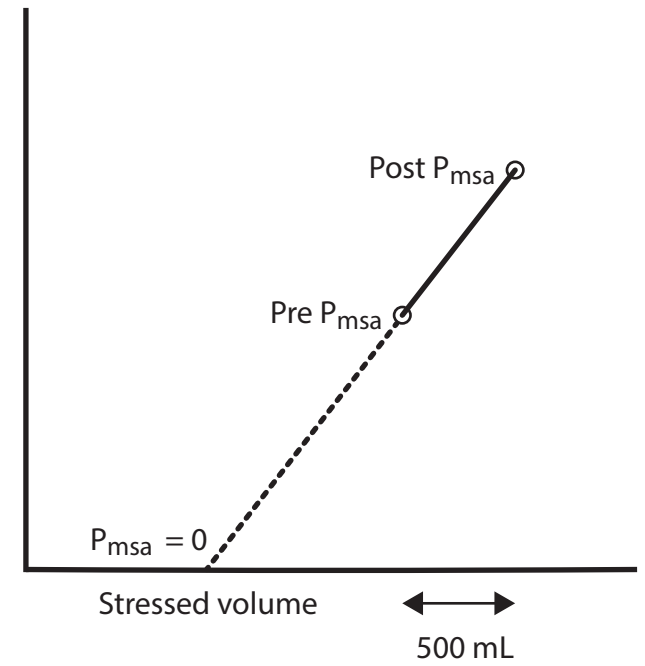

Supplement: Supplementary file 2 — Additional file 2. Illustration of haemodynamic physiology concepts. A Cardiac output is determined by the intersection of the venous return and cardiac function curves, indicated in the graph for the positions pre and post the administration of a volume bolus. The corresponding change in venous return pressure gradient (VRdP) over the change in the analogue mean systemic filling pressure (Pmsa) describes the efficiency of the volume bolus to increase cardiac output (Evol). B Volume pressure curve of the left ventricle indicating the slope of the effective arterial elastance given by the end-systolic blood pressure (ESP) over the stroke volume (SV). C Analogue mean systemic filling pressure before (Pre Pmsa) administration of a known quantity of volume (500 mL) and the resulting increase in filling pressure (Post Pmsa). The line connecting these two observations was extrapolated to a Pmsa of zero, indicating the stressed volume, i.e., the volume that starts to generate an elastic recoil pressure within the vasculature [file 40635_2024_657_MOESM2_ESM.pdf]
